# Off-Grid Underwater Acoustic Source Direction-of-Arrival Estimation Method Based on Iterative Empirical Mode Decomposition Interval Threshold

**DOI:** 10.3390/s24175835

**Published:** 2024-09-08

**Authors:** Chuanxi Xing, Guangzhi Tan, Saimeng Dong

**Affiliations:** 1 College of Electrical and Information Technology, Yunnan Minzu University, Kunming 650504, China; tanguangzhi_ymu@hotmail.com (G.T.); dongsaimeng@163.com (S.D.); 2 Yunnan Key Laboratory of Unmanned Autonomous System, Kunming 650504, China

**Keywords:** hydrophone arrays, DOA, empirical mode decomposition, Bayesian learning algorithm

## Abstract

To solve the problem that the hydrophone arrays are disturbed by ocean noise when collecting signals in shallow seas, resulting in reduced accuracy and resolution of target orientation estimation, a direction-of-arrival (DOA) estimation algorithm based on iterative EMD interval thresholding (EMD-IIT) and off-grid sparse Bayesian learning is proposed. Firstly, the noisy signal acquired by the hydrophone array is denoised by the EMD-IIT algorithm. Secondly, the singular value decomposition is performed on the denoised signal, and then an off-grid sparse reconstruction model is established. Finally, the maximum a posteriori probability of the target signal is obtained by the Bayesian learning algorithm, and the DOA estimate of the target is derived to achieve the orientation estimation of the target. Simulation analysis and sea trial data results show that the algorithm achieves a resolution probability of 100% at an azimuthal separation of 8° between adjacent signal sources. At a low signal-to-noise ratio of −9 dB, the resolution probability reaches 100%. Compared with the conventional MUSIC-like and OGSBI-SVD algorithms, this algorithm can effectively eliminate noise interference and provides better performance in terms of localization accuracy, algorithm runtime, and algorithm robustness.

## 1. Introduction

In recent years, with the increasing demand for marine resource development, environmental protection, and national defense, hydrophones have been widely used in various fields such as oil and gas exploration, subsea geological surveys, marine ecological monitoring, noise pollution control, and submarine detection [1]. However, despite significant advancements in hydrophone technology, many challenges remain. The noise in marine environments is complex and variable, comprising both natural sources (such as waves, marine life, and rain) and artificial sources (such as ships and military sonar) [2]. The non-stationary and complex characteristics of this noise greatly increase the difficulty of signal processing. Furthermore, marine noise often consists of a mixture of multiple noise sources, forming a complex noise field that further complicates the extraction and identification of target signals. Against this backdrop, it is crucial to develop effective denoising algorithms and methods to improve the positioning accuracy of hydrophone signals and address the challenges posed by complex underwater environments and noise interference [3].

In the field of underwater acoustic DOA estimation, hydrophone arrays have been widely employed for source localization [4]. Traditional spatial spectrum estimation algorithms include Conventional Beamforming (CBF) [5,6], Minimum Variance Distortionless Response (MVDR), and subspace algorithms with super-resolution capabilities such as MUSIC and ESPRIT [7,8]. However, in unfavorable environments such as low signal-to-noise ratios, the performance of these algorithms in estimating the target orientation can be significantly reduced. In order to enhance the precision of location determination, a number of sparse signal processing algorithms, including Bayesian inference (SBI), have been put forth as potential solutions [9]. Nevertheless, these methods rely on predefined spatial sampling grids. Despite the high grid density, the fact that actual signal sources do not precisely align with these grid points limits the angle resolution. To address this issue, gridless algorithms and algorithms that incorporate off-grid errors have been widely studied. The authors of [10] proposed a gridless convex optimization problem to address this issue by recovering the covariance matrix of a virtual array. The authors of [11] introduced a perturbed Sparse Signal Representation (SSR) model, incorporating bias parameters into the DOA estimation framework to address these challenges. The authors of [12] tackled the angle resolution limitation caused by sources not precisely aligning with grid points by constructing and optimizing a co-array tensor embedded with displacement information. The authors of [13] introduced the off-grid sparse Bayesian inference (OGSBI) algorithm, which incorporates off-grid errors into the array’s manifold matrix, thereby avoiding the substantial computational burden associated with mesh refinement and overcoming the angle resolution limitations.

The non-stationarity of underwater acoustic signals coupled with the presence of interfering noises renders traditional stationary signal processing methods inadequate. To address this, algorithms such as empirical mode decomposition (EMD) and wavelet transform are utilized due to their proficient handling of non-stationary signals. In 1998, Hilbert Huang et al. proposed an adaptive signal processing method, empirical mode decomposition (EMD), for the analysis of non-linear non-stationary time series, which is based on the characteristic time scale of the signal itself. The method does not require the setting of a basis function. The intrinsic modal function is generated adaptively based on the analyzed signal [14]. In the studies by [15,16], ensemble empirical mode decomposition (EEMD) and modified ensemble EMD (MEEMD) algorithms have been proposed to reduce the aliasing problem in empirical modal decomposition by adding noise to the original data and also to extend it to the field of high-dimensional processing. In the study by [17], an iterative EMD interval thresholding denoising algorithm is proposed, which is based on the idea of EMD with translation-invariant wavelet thresholding, and builds different noisy versions of the original signal by changing the position of the first intrinsic mode function (IMF) sample through multiple iterations, and then obtains multiple denoised versions of the signal by the IMF thresholding method and averaging them to enhance the denoising ability.

Specifically, the contributions of our work can be summarized as follows:

- The algorithm leverages EMD for the adaptive decomposition of non-stationary signals into their respective IMFs. To overcome EMD’s limitations in handling complex noise, IIT is applied to iteratively threshold the IMFs, effectively filtering out low-energy noise. This combined approach significantly enhances noise suppression, ensuring accurate DOA estimation even under low signal-to-noise ratio (SNR) conditions.
- The integration with OGSBI further optimizes signal reconstruction, enhancing target resolution and addressing grid mismatch issues, which is particularly effective when dealing with closely spaced signal sources.
- The use of singular value decomposition (SVD) reduces computational complexity, making this method not only more accurate than traditional algorithms but also more efficient in real-time applications.

The paper is structured as follows: Section 2 introduces the background of EMD (empirical mode decomposition) and discusses the traditional DOA (direction of arrival) estimation method in detail. It also establishes the signal reception model and elaborates on the principles and processes of the EMDIIT-OGSBI algorithm. Section 3 compares the EMDIIT-OGSBI algorithm with the MUSIC algorithm and the OGSBI-SVD algorithm through simulation analysis and analyzes the superiority of the algorithm in this paper. Section 4 presents the source of the experimental data from the sea trial and uses it to validate the algorithm. The conclusions are given in Section 5.

## 2. Methods

### 2.1. Signal Processing Model for Hydrophone Arrays

**Figure 1:**
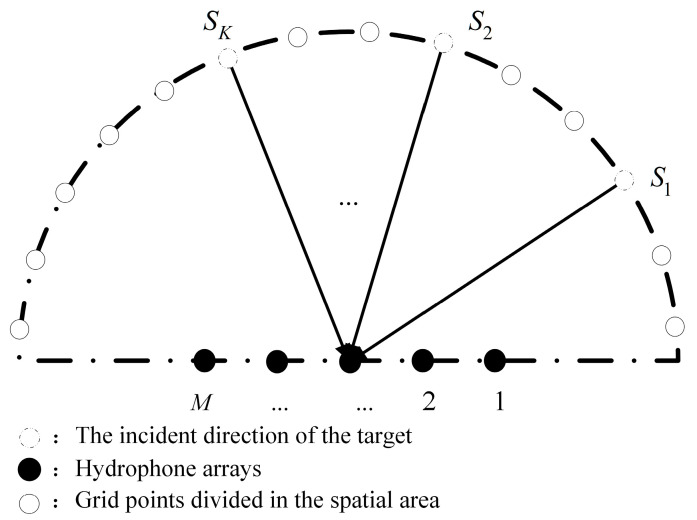
Model diagram of array received signal.

Multiple hydrophone array elements arranged in space according to a certain geometric position constitute a base array, which has better directivity than a single array element. It is assumed that there are *M* uniformly deployed hydrophone arrays for hydroacoustic signal localization [18], with neighboring arrays spaced d=λ/2 and λ being the signal wavelength. Under the plane wave assumption [19], the hydrophone base array with an array streamlines of $a(\theta )\in {\mathbb{C}}^{M\times 1}$ receives K far-field uncorrelated narrowband signals. As shown in Figure 1, the incident azimuth is $\Theta_{s}=[\theta_{1},\theta_{2},\cdots ,\theta_{K}^{T}]$, and ${\left[\cdot \right]}^{T}$ denotes transposition, where $\theta_{K}$ denotes the orientation of the Kth signal. The orientation of the hydrophone array is measured relative to the zero angle, which corresponds to the broadside angle of the array. At the instantaneous moment t, the base array output vector $x(t)\in {\mathbb{C}}^{M\times 1}$ is expressed as [20]

(1) $$x(t)=A(\Theta_{s})s(t)+n(t)$$

where

$x(t)={\left[x_{1}(t),x_{2}(t),\cdots ,x_{M}(t)\right]}^{T}\in {\mathbb{C}}^{M\times 1}$ is the array signal,
$s(t)={\left[s_{1}(t),s_{2}(t),\cdots ,s_{K}(t)\right]}^{T}\in {\mathbb{C}}^{K\times 1}$ is the source signal,
$n(t)={\left[n_{1}(t),n_{2}(t),\cdots ,n_{M}(t)\right]}^{T}\in {\mathbb{C}}^{M\times 1}$ is the array noise,
$A\left(\Theta_{s}\right)=[a(\theta_{1}),a(\theta_{2}),\cdots ,a(\theta_{K})]\in {\mathbb{C}}^{M\times K}$ is the array guidance vector, and $a_{m}(\theta_{k})$ is the array stream vector for the kth signal received by the mth array element, with
  (2) $$a\left(\theta_{k}\right)=\left[\begin{array}{c} 1\\ e^{-j\frac{2\pi }{\lambda }d\sin\theta_{k}}\\ \vdots \\ e^{-j\frac{2\pi }{\lambda }(M-1)d\sin\theta_{k}} \end{array}\right]$$

If each source signal has T snapshots, the model for multiple snapshots can be written as

(3) $$X=A\left(\Theta_{s}\right)S+N$$

where

$X=[x(1),x(2),\cdots ,x(T)]\in {\mathbb{C}}^{M\times T}$ is the signal vector received by the array,
$S=[s(1),s(2),\cdots ,s(T)]\in {\mathbb{C}}^{K\times T}$ is the signal vector emitted by the source, and
$N=[n(1),n(2),\cdots ,n(T)]\in {\mathbb{C}}^{M\times T}$ is the noise vector to which the array is subjected.

### 2.2. Iterative EMD Interval Thresholding Methodology

The EMD-IIT denoising algorithm combines EMD with a translation-invariant wavelet thresholding method [21], and the section below describes the specific principles involved in this denoising algorithm.

#### 2.2.1. Empirical Mode Decomposition

The basic idea of EMD [22] is that for all signals, the time series can be decomposed into a finite number of IMFs with different characteristic scales, each representing a characteristic oscillation in a time scale and with local orthogonality and adaptive properties. In the analysis of non-stationary signals, the interference of the same frequency components between different components over time can be effectively avoided.

In the signal model established above for array reception, where the array element received signal is $X\in {\mathbb{C}}^{M\times T}$, we perform EMD of each array element signal separately to obtain IMFs and residuals for each row vector, as follows:

(4) $$X=\left[\begin{array}{c} x_{1}(t)\\ x_{2}(t)\\ \vdots \\ x_{m}(t) \end{array}\right]=\left[\begin{array}{c} \overset{L}{\underset{i=1}{\sum }}IMF_{1i}(t)+r_{1L}(t)\\ \overset{L}{\underset{i=1}{\sum }}IMF_{2i}(t)+r_{2L}(t)\\ \vdots \\ \overset{L}{\underset{i=1}{\sum }}IMF_{mi}(t)+r_{mL}(t) \end{array}\right]$$

where $r_{mL}(t)$ is the residual, which is a slowly varying function of the non-zero mean with only a few extreme values. Here, i=1,⋯,L, m=1,⋯,M, and t=1,⋯,T.

The EMD algorithm proceeds as follows, taking as an example the first array received signal $x_{1}(t)\in {\mathbb{C}}^{1\times T}$ of the array received signal as the input signal:

(1) Find the local maxima and minima of a, obtain the sequence of maxima and minima, and interpolate the maxima and minima, respectively, to obtain the upper and lower envelopes of $x_{1}(t)$. The two envelopes are asymmetric to each other.
(2) Average the maxima and minima envelopes, and obtain the envelope mean $m_{1}(t)$. Subtract the envelope mean from the original signal to obtain the first component $h_{1}(t)=x_{1}(t)-m_{1}(t)$.
(3) Use $h_{1}(t)$ as the input and repeat steps 1 and 2 to obtain $h_{2}(t)$, iterating continuously. Use the standard deviation of the two adjacent decomposition components as the criterion for stopping the iteration, which is generally taken as 0.2≤S≤0.3. At this point, obtain $h_{p}(t)$. Calculate S as $S=\sum_{t=1}^{T}\frac{{\left|h_{p-1}(t)-h_{p}(t)\right|}^{2}}{{\left(h_{p-1}(t)\right)}^{2}}$.
(4) $h_{p}(t)$ is the first IMF, noted as $IMF_{11}(t)$, and subtract the input signal from $IMF_{11}(t)$ to obtain the residual quantity $r_{11}(t)=x_{1}(t)-IMF_{11}(t)$.
(5) Use $r_{11}(t)$ as the new input signal and re-execute steps 1 to 5 to obtain a new residual $r_{12}(t)$ and a second $IMF_{12}(t)$, and so on, to obtain $IMF_{13}(t),IMF_{14}(t),\cdots ,IMF_{1L}(t)$. At this point, the residual $r_{1L}(t)$ becomes a monotonic function and cannot be decomposed into IMF again. The final result is $x_{1}(t)=\sum_{i=1}^{L}IMF_{1i}(t)+r_{1L}(t)$.

**Figure 2:**
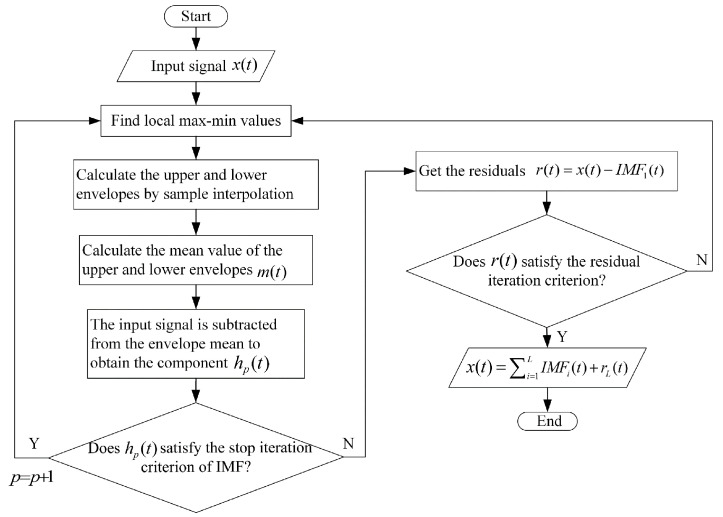
Iteration flow chart of EMD algorithm.

The iterative flow chart of the EMD algorithm is shown in Figure 2.

#### 2.2.2. Wavelet Threshold Denoising

Wavelet threshold denoising has the feature of suppressing the useless part of the signal and enhancing the useful part [23,24]. The following describes the principle of wavelet threshold denoising:

The following noise signal y(t) is given by

(5) y(t)=x(t)+σn(t)

where x(t) is the original signal, n(t) is the white noise signal following a standard Gaussian distribution, and σ is the noise variance. The wavelet threshold denoising method starts with the selection of a suitable wavelet basis W, and the discrete wavelet transform (DWT) is obtained as

(6) c=Wy

where $c=[c_{1},c_{2},\cdots ,c_{T}]$ is the wavelet decomposition coefficient. Then, a threshold is set for quantization, i.e., λ=σC, where *C* is a constant. The basic principle of wavelet thresholding is to set all wavelet coefficients below that threshold to zero, and to keep those above that threshold directly or to process them accordingly. Currently, the most common thresholding functions are the hard and soft threshold values proposed by Donoho [25], which are defined as

(7) $$\rho_{\lambda }(v)=\{\begin{array}{cc} v, & \left|v\right|>\lambda \\ 0, & \left|v\right|\leq \lambda \end{array}$$

and

(8) $$\rho_{\lambda }(v)=\{\begin{array}{ll} \mathrm{sgn}(v)(\left|v\right|-\lambda ), & \left|v\right|>\lambda \\ 0, & \left|v\right|\leq \lambda \end{array}$$

For the selection of the threshold value, the common threshold formula is $\lambda =\sigma \sqrt{2\ln T}$, which guarantees a high probability that all noisy components will have a low amplitude. Here, T is the length of the sampled signal, and σ is the standard deviation of the noisy signal, which is estimated as [26]

(9) $$\sigma =\frac{\mathrm{median}\left(\left|v_{i}\right|\right)}{0.6745}$$

After processing with hard or soft thresholding, the denoised signal is obtained by inverting the processed wavelet coefficients, as follows:

(10) $$\tilde{y}=W^{T}\tilde{c}$$

where $\tilde{c}=[\rho_{\lambda }(v_{1}),\rho_{\lambda }(v_{2}),\cdots ,\rho_{\lambda }(v_{T})]$.

#### 2.2.3. Implementation of Iterative EMD Interval Thresholding

The IMF threshold denoising method in this paper is related to the wavelet threshold denoising method. Wavelet denoising is the thresholding of wavelet components, whereas in the EMD case, IMF thresholding is performed on samples from each IMF. However, the fact is that the noise contained in each IMF is colored, i.e., each mode has a different energy in it. In this sense, EMD denoising is most closely related to wavelet denoising of signals corrupted by colored noise, where the threshold must be scale-dependent, and by adapting the threshold function to the nature of each IMF, threshold denoising of the IMF obtained by EMD can locally exclude the low-energy IMF parts disturbed by high noise. Based on the idea of wavelet thresholding, the IMF threshold is given by

(11) $${\tilde{h}}_{IMF_{i}}(t)=\{\begin{array}{ll} h_{IMF_{i}}(t), & \left|h_{IMF_{i}}(t)\right|>\lambda_{i}\\ 0, & \left|h_{IMF_{i}}(t)\right|\leq \lambda_{i} \end{array}$$

for hard thresholding and given by

(12) $${\tilde{h}}_{IMF_{i}}(t)=\{\begin{array}{ll} \mathrm{sgn}\left(h_{IMF_{i}}(t)\right)\;\left(\left|h_{IMF_{i}}(t)\right|-\lambda_{i}\right), & \left|h_{IMF_{i}}(t)\right|>\lambda_{i}\\ 0, & \left|h_{IMF_{i}}(t)\right|\leq \lambda_{i} \end{array}$$

for soft thresholding. In both thresholding cases, we can see that the thresholds are also different for the different IMFs, where ${\tilde{h}}_{IMF_{i}}(t)$ represents the ith thresholded IMF. The thresholds we study become multiples of the independent thresholds for each IMF, i.e., $\lambda_{k}=C\sqrt{E_{k}2\ln T}$, where $E_{k}$ represents the energy of the kth IMF, which is given by

(13) $$E_{k}=\frac{E_{1}^{2}}{\beta }\rho^{-k}$$

where $E_{1}^{2}$ is the energy of the first IMF. According to [27], the values of parameters β and ρ are 0.719 and 2.01.

The reconstruction of the denoised signal is given by

(14) $$\hat{x}(t)=\sum_{k=M_{1}}^{M_{2}}{\tilde{h}}_{IMF_{i}}(t)+\sum_{k=M_{2}+1}^{L}h_{IMF_{i}}(t)$$

In particular, the addition of the $M_{1}$ and $M_{2}$ parameters gives us more flexibility in excluding noisy low-order IMFs and in selecting thresholds for higher-order IMFs. Regarding the parameter $M_{1}$, it is calculated by conventional EMD denoising [28,29,30]. The denoised signal must be reconstructed from IMFs of order J and higher; in other words, the energy of the noise is lower than the energy of the signal in IMFs of order J and higher. The best choice for the $M_{1}$ parameter is

(15) $$M_{1}=\max(1,J-2)$$

And the best choice for the parameter is $M_{2}=L-2$.

For an independent IMF sample amplitude, it is not possible to infer whether it corresponds to a noisy or useful signal. However, it is possible to guess whether the signal within this interval is noise-dominated or signal-dominated, based on the single extreme value $h_{IMF_{i}}(r_{IMF_{i}}^{\left(j\right)})$ corresponding to the interval $t_{IMF_{i}}^{\left(j\right)}=[t_{IMF_{i}}^{\left(j\right)}\;t_{IMF_{i}}^{\left(j+1\right)}]$ adjacent to the over-zero point, where j=1,2⋯,T and i=1,2⋯,L. If a strong signal is present within this interval, the absolute value of the extreme value will exceed the threshold; conversely, if the signal is small, the absolute value of the extreme value will be below the threshold. Thus, the modified hard and soft thresholds, referred to as the EMD interval threshold (EMD-IT), are

(16) $${\tilde{h}}_{IMF_{i}}\left(t_{IMF_{i}}^{\left(j\right)}\right)=\left\{ \begin{array}{ll} h_{IMF_{i}}\left(t_{IMF_{i}}^{\left(j\right)}\right), & \left|h_{IMF_{i}}\left(r_{IMF_{i}}^{\left(j\right)}\right)\right|>\lambda_{i}\\ 0, & \left|h_{IMF_{i}}\left(r_{IMF_{i}}^{\left(j\right)}\right)\right|\leq \lambda_{i} \end{array}\right.$$

and

(17) $${\tilde{h}}_{IMF_{i}}\left(t_{IMF_{i}}^{\left(j\right)}\right)=\left\{ \begin{array}{ll} h_{IMF_{i}}\left(t_{IMF_{i}}^{\left(j\right)}\right)\frac{\left|h_{IMF_{i}}\left(r_{IMF_{i}}^{\left(j\right)}\right)\right|-\lambda_{i}}{h_{IMF_{i}}\left(r_{IMF_{i}}^{\left(j\right)}\right)}, & \left|h_{IMF_{i}}\left(r_{IMF_{i}}^{\left(j\right)}\right)\right|>\lambda_{i}\\ 0, & \left|h_{IMF_{i}}\left(r_{IMF_{i}}^{\left(j\right)}\right)\right|\leq \lambda_{i} \end{array}\right.$$

where $h_{IMF_{i}}\left(t_{IMF_{i}}^{\left(j\right)}\right)$ represents the sample values from interval $t_{IMF_{i}}^{\left(j\right)}$ to $t_{IMF_{i}}^{\left(j+1\right)}$ in the ith IMF.

**Figure 3:**
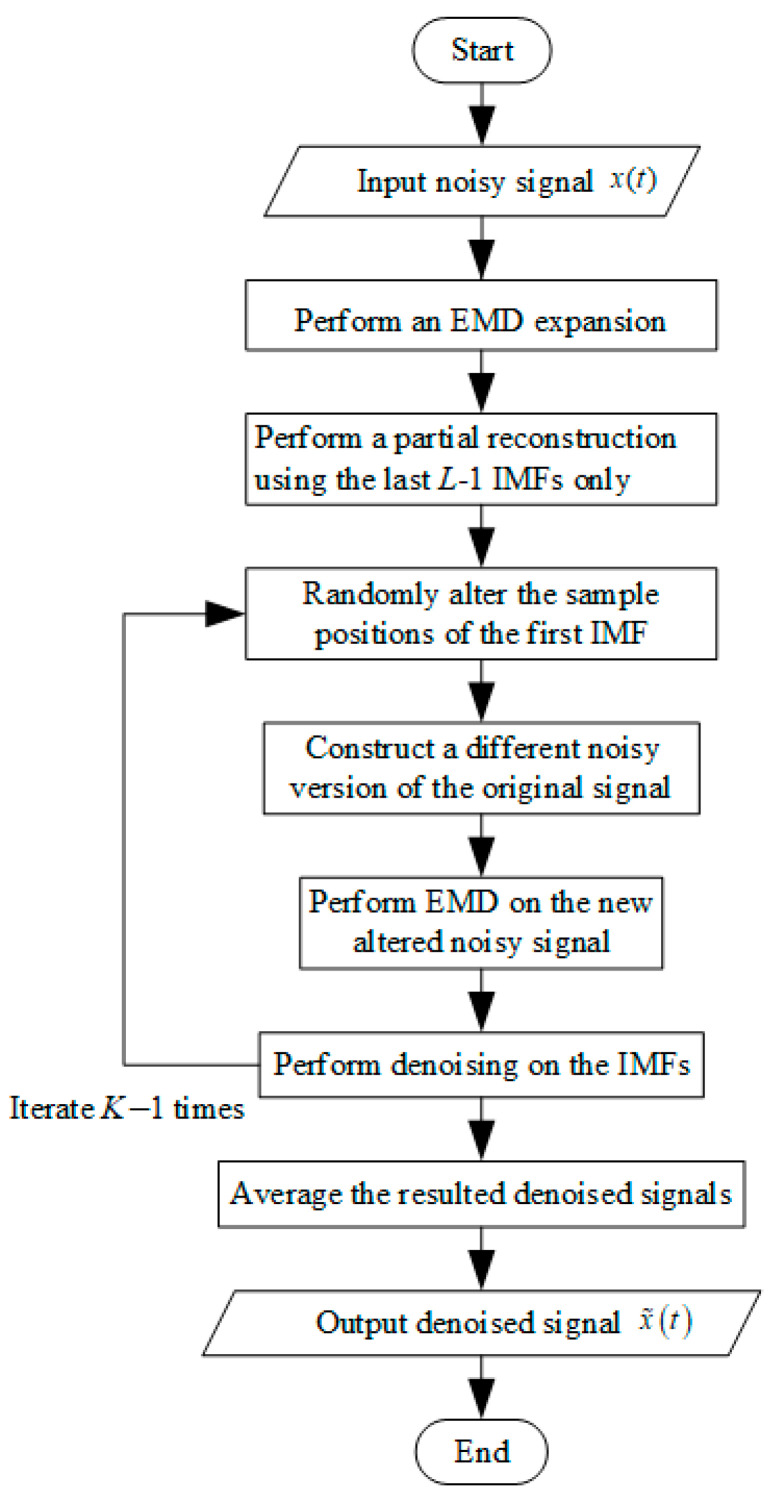
Flowchart of EMD-IIT algorithm.

Based on the idea of translation-invariant wavelet thresholding [28], multiple denoised versions of the signal are obtained by iteration, and their denoising power is enhanced by averaging them. In the EMD case, different denoised versions of the noisy signal can only be obtained by thresholding different versions of the IMF. We know that under Gaussian white noise conditions, the first IMF is mainly noise; in other words, it contains more noise than the others. By changing the position of the first IMF sample and then adding the newly generated noise signal to the sum of the remaining IMFs, we can obtain a different noisy version of the original signal. In fact, when the first IMF contains only noise, the total noise variance of the newly generated noise signal is the same as that of the original noise signal. Figure 3 shows the flowchart of the algorithm referred to as iterative EMD interval thresholding (EMD-IIT), in the following steps:

(1) EMD expansion of the initial noisy signal x(t).
(2) Local reconstruction using only the last L-1 IMFs, $x_{p}\left(t\right)=\sum_{i=2}^{L}IMF_{i}\left(t\right)$.
(3) Randomly changing the sample position of the first IMF, $IMF_{1}^{\left(a\right)}\left(t\right)=\mathrm{ALTER}\left(IMF_{1}\left(t\right)\right)$.
(4) Constructing a different noisy version of the original signal, $x^{\left(a\right)}\left(t\right)=x_{p}\left(t\right)+IMF_{1}^{\left(a\right)}\left(t\right)$.
(5) Performing EMD processing on the newly obtained noisy signal $x^{\left(a\right)}\left(t\right)$.
(6) The denoised version of the original signal, ${\tilde{x}}_{1}\left(t\right)$ of x, is obtained by denoising the IMFs of the newly obtained noisy signal $x^{\left(a\right)}\left(t\right)$ by Formula (16) or Formula (17).
(7) Iterating Q−1 more times in steps 3–6 (typically, Q is set to 20) to obtain q denoised versions of *x*, i.e., ${\tilde{x}}_{1},{\tilde{x}}_{2},\cdots ,{\tilde{x}}_{Q}$.
(8) Averaging of the noise-reduced signal, $\tilde{x}\left(t\right)=\left(1/Q\right)\sum_{q=1}^{Q}{\tilde{x}}_{q}\left(t\right)$.

### 2.3. The Algorithm for Iterative EMD Interval Thresholding and Off-Grid Sparse Bayesian Learning

**Figure 4:**
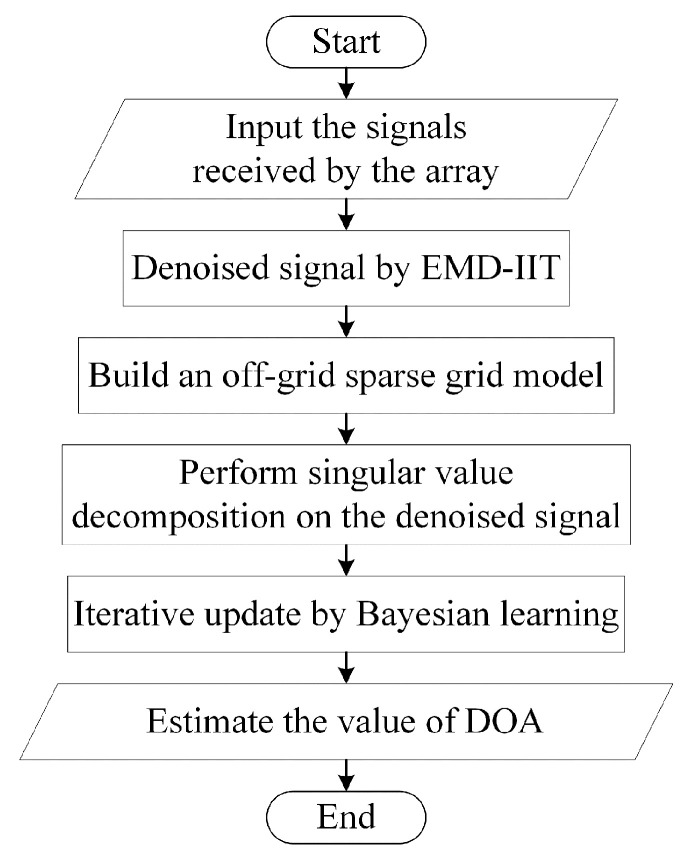
Flowchart of algorithm for iterative EMD interval thresholding and off-grid sparse Bayesian learning.

The DOA estimation algorithm in this paper consists of the following parts: the first part is to perform EMD-IIT denoising on the noisy signal received by each array element to obtain the signal with noise interference removed; the second part is to specify the signal space by building an off-grid sparse grid model and to decompose the denoised signal into singular values to further reduce the sensitivity to noise; and the third part is to learn by Bayesian continuous iteration, updating the hyperparameters, and finally reaching a state of convergence to obtain the DOA estimates. The overall framework of the algorithm is shown in Figure 4.

#### 2.3.1. EMD-IIT Denoising

According to Formula (3), we obtain the noise-bearing signal received by the hydrophone arrays, i.e., $X=A(\Theta_{s})S+N$, where $X\in {\mathbb{C}}^{M\times T}$ is the signal of T snapshots. Here, with the EMD-IIT algorithm above, we denoise the noisy signal. Since each array is subjected to EMD-IIT, the signal matrix received by the mth array element is defined as $X_{m}\in {\mathbb{C}}^{1\times T}$ for ease of calculation. We write the noise-bearing signal in the following form:

(18) $$X=\left[\begin{array}{c} X_{1}\\ X_{2}\\ \vdots \\ X_{M} \end{array}\right]$$

Then, the EMD-IIT algorithm is used to denoise each row of the noisy signal separately to obtain the denoised signal $\hat{X}=[{\hat{X}}_{1},{\hat{X}}_{2},\cdots ,{\hat{X}}_{m}]$. Then, the new array signal vector after removing the noise is

(19) $$\hat{X}=\hat{A}(\Theta_{s})\hat{S}$$

where $\hat{X}$ is the vector signal of an M×T dimension.

#### 2.3.2. Off-Grid Sparse Model and Singular Value Decomposition

The spatial angle range [−π/2,π/2] is uniformly divided into N grid points, with each point representing a potential incident direction, such as $\tilde{\Theta }=\{{\tilde{\theta }}_{1},{\tilde{\theta }}_{2},\cdots ,{\tilde{\theta }}_{N}\}$, and K<M≪N. From this, the grid interval $r=\{{\tilde{\theta }}_{2}-{\tilde{\theta }}_{1},{\tilde{\theta }}_{3}-{\tilde{\theta }}_{2},\cdots ,{\tilde{\theta }}_{N}-{\tilde{\theta }}_{N-1}\}$ can be determined. It is evident that the target orientation can be reinterpreted as an overcomplete sparse representation across the N divided grid points. In the off-grid sparse model, there is an issue where the target incidence direction does not align perfectly with the grid points, resulting in a mismatch, represented as $\theta_{k}\notin \{{\tilde{\theta }}_{1},{\tilde{\theta }}_{2},\cdots ,{\tilde{\theta }}_{N}\}$; generally a denser grid point can reduce the error, but this does not contain all possible incidence directions and increases the computational effort. To address this issue, an off-grid error is incorporated into the array’s prevalence matrix by performing a first-order Taylor expansion between two neighboring grid points, allowing the steering vector to be approximated as

(20) $$\varphi \left(\theta_{k}\right)\approx a({\tilde{\theta }}_{n_{k}})+b({\tilde{\theta }}_{n_{k}})(\theta_{k}-{\tilde{\theta }}_{n_{k}})$$

where ${\tilde{\theta }}_{n_{k}}$ denotes the nearest grid point to $\theta_{k}$, $n_{k}\in \{1,2,\cdots ,N\}$, and $b({\tilde{\theta }}_{n_{k}})=a^{\prime }({\tilde{\theta }}_{n_{k}})$. By letting $\beta ={\left[\beta_{1},\beta_{2},\cdots ,\beta_{N}\right]}^{T}\in {\left[-r/2,r/2\right]}^{N}$, the grid error can be expressed as

(21) $$\beta_{n}=\left\{ \begin{array}{ll} \theta_{k}-{\tilde{\theta }}_{n_{k}}, & \hat{S}\neq 0,n=n_{k}\\ 0, & \hat{S}=0,n\neq n_{k} \end{array}\right.$$

According to Formula (20), the overcomplete array popularity matrix can be expressed as

(22) $$\Phi ={\hat{A}}_{\tilde{\theta }}+B\mathrm{diag}\{\beta \}$$

Considering the case where the background noise has been filtered out above, we have $\hat{X}=\Phi \hat{S}$, where K≤T and $\mathrm{Rank}(\hat{X})\leq \mathrm{Rank}(\hat{S})\leq K$. Let $V=[V_{1}\;V_{2}]$, where $V_{1}$ and $V_{2}$ are matrices consisting of the first K columns of V and the remaining T−K columns, respectively. By singular value decomposition, we obtain matrices $\hat{X}V=[{\hat{X}}_{\mathrm{SV}}\;\hat{X}V_{2}]$ containing all signal information, where ${\hat{X}}_{\mathrm{SV}}=\hat{X}V_{1}\in {\mathbb{C}}^{M\times K}$, and where the first part ${\hat{X}}_{\mathrm{SV}}$ retains most of the signal information and is used in the signal reconstruction process later, and the second part is discarded. Let ${\hat{S}}_{\mathrm{SV}}=\hat{S}V_{1}$; then, we can represent the signal after singular value decomposition as

(23) $${\hat{X}}_{\mathrm{SV}}=\Phi {\hat{S}}_{\mathrm{SV}}$$

where the signal ${\hat{S}}_{\mathrm{SV}}$ still has joint sparsity [29].

#### 2.3.3. Sparse Bayesian Inference

The Bayesian inference method is used for the estimation of hydroacoustic target orientation, and the optimal estimate can be obtained. Assuming that the noise signal obeys the complex Gaussian distribution, the following likelihood function of ${\hat{X}}_{\mathrm{SV}}$ is given by

(24) $$p\left({\hat{X}}_{\mathrm{SV}}|{\hat{S}}_{\mathrm{SV}};\alpha_{0}\right)=\mathrm{CN}\left({\hat{X}}_{\mathrm{SV}}|\Phi {\hat{S}}_{\mathrm{SV}},\alpha_{0}^{-1}\right)$$

where CN represents the complex Gaussian distribution, $\alpha_{0}=\sigma^{-2}$, where $\sigma^{2}$ is the noise variance and $\alpha_{0}$ is usually unknown, which is assumed to obey the Gamma prior distribution.

Let the prior probability of the sparse signal ${\hat{S}}_{\mathrm{SV}}$ be

(25) $$p\left({\hat{S}}_{\mathrm{SV}}|\gamma \right)=\mathrm{CN}\left({\hat{S}}_{\mathrm{SV}}|0,\Gamma \right)$$

where γ is a set of hyperparameters, $\gamma ={\left[\gamma_{1},\gamma_{2},\cdots ,\gamma_{N}\right]}^{T}$, which represents the source signal power incident to the array in each direction and also affects the sparsity of the sparse signal. Here, Γ=diag(γ) represents the covariance matrix of the sparse signal.

Combining the prior information and the likelihood function, the joint probability density function is obtained as

(26) $$\begin{array}{c} p\left({\hat{S}}_{\mathrm{SV}},{\hat{X}}_{\mathrm{SV}},\alpha_{0},\gamma ,\beta \right)\\ =p\left({\hat{X}}_{\mathrm{SV}}|{\hat{S}}_{\mathrm{SV}},\alpha_{0},\beta \right)p\left({\hat{S}}_{\mathrm{SV}}|\gamma \right)p\left(\alpha_{0}\right)p\left(\gamma \right)p\left(\beta \right) \end{array}$$

The posterior probability density function of ${\hat{S}}_{\mathrm{SV}}$ can be obtained from Bayesian inference as

(27) $$\begin{array}{l} p\left({\hat{S}}_{\mathrm{SV}}|{\hat{X}}_{\mathrm{SV}},\alpha_{0},\gamma ,\beta \right)\\ =\frac{p\left({\hat{X}}_{\mathrm{SV}}|{\hat{S}}_{\mathrm{SV}},\alpha_{0},\beta \right)p\left({\hat{S}}_{\mathrm{SV}}|\gamma \right)}{p\left({\hat{X}}_{\mathrm{SV}}|\alpha_{0},\gamma ,\beta \right)}\\ =\mathrm{CN}\left({\hat{S}}_{\mathrm{SV}}|\mu ,\Sigma \right) \end{array}$$

where the posterior mean μ and posterior covariance matrix Σ of the sparse signal are given separately by

(28) $$\mu =\alpha_{0}\Sigma \Phi^{H}{\hat{X}}_{\mathrm{SV}}$$

and

(29) $$\Sigma ={\left(\alpha_{0}\Phi^{H}\Phi +\Gamma^{-1}\right)}^{-1}$$

where μ and Σ are functions with respect to the three hyperparameters $\alpha_{0}$, γ, and β. We use a maximum posterior probability criterion to maximize the probability $p\left(\alpha_{0},\gamma ,\beta |{\hat{X}}_{\mathrm{SV}}\right)$, which leads to the derivation of two parameters $\alpha_{0}$ and γ. These two parameters are given by

(30) $$\alpha_{0}^{\mathrm{new}}=\frac{TM+c-1}{d+\sum_{t=1}^{T}{\left\|{\hat{X}}_{\mathrm{SV}}\left(t\right)-\Phi \mu \left(t\right)\right\|}_{2}^{2}+T\mathrm{tr}(\Phi^{H}\Sigma \Phi )}$$

and

(31) $$\gamma_{n}^{\mathrm{new}}=\frac{-T+\sqrt{T^{2}+4\rho \sum_{t=1}^{T}{\left[\Xi_{t}\right]}_{nn}}}{2\rho }\;,\;n=1,2,\cdots ,N$$

where $\Xi_{t}≜\mu (t){\left(\mu (t)\right)}^{H}$, c,d→0, and ρ is a positive constraint taking small values.

The grid error β determines the accuracy of the target orientation estimation. We can use the expectation maximization criterion to find the grid error so that the expectation $E\left\{\ln\left[p\left({\hat{X}}_{\mathrm{SV}}|{\hat{S}}_{\mathrm{SV}},\alpha_{0},\beta \right)p\left(\beta \right)\right]\right\}$ is maximized, which is equivalent to minimizing $E\left\{{\left\|{\hat{X}}_{\mathrm{SV}}-\Phi {\hat{S}}_{\mathrm{SV}}\right\|}_{2}^{2}\right\}$, as follows:

(32) $$\begin{array}{ll} E\left\{{\left\|{\hat{X}}_{\mathrm{SV}}-\Phi {\hat{S}}_{\mathrm{SV}}\right\|}_{2}^{2}\right\} & =E\left\{{\left\|{\hat{X}}_{\mathrm{SV}}-\left({\hat{A}}_{\tilde{\theta }}+B\mathrm{diag}\left\{\beta \right\}\right){\hat{S}}_{\mathrm{SV}}\right\|}_{2}^{2}\right\}\\ & =\beta^{T}P\beta -2v^{T}\beta +C \end{array}$$

where C is a constant depending on β. P is a semi-positive definite matrix whose expression is given by

(33) $$P=ℜ\left\{\bar{B^{H}B}⊙\left(\mu \mu^{H}+\Sigma \right)\right\}$$

(34) $$v=ℜ\left\{\mathrm{diag}\left(\bar{\mu }\right)B^{H}\left({\hat{X}}_{\mathrm{SV}}-{\hat{A}}_{\tilde{\theta }}\mu \right)-\mathrm{diag}\left(B^{H}{\hat{A}}_{\tilde{\theta }}\Sigma \right)\right\}$$

The angle correction vector is obtained by the above derivation as [30]

(35) $$\beta^{\mathrm{new}}=\arg\underset{\beta \in {\left[-\frac{r}{2},\frac{r}{2}\right]}^{N}}{\min}\left\{\beta^{T}P\beta -2v^{T}\beta \right\}$$

Next, we obtain the expression for β by taking the derivative of β by Formula (32) and setting it to zero as

(36) $${\tilde{\beta }}_{n}=\frac{v_{n}-{\left(P_{n}\right)}_{-n}^{T}\beta_{-n}}{P_{nn}}$$

where $\beta_{-n}$ is β without the nth entry for a vector β. By means of the constraint $\beta_{n}\in [-r/2,r/2]$, we have

(37) $${\tilde{\beta }}_{n}^{\mathrm{new}}=\{\begin{array}{ll} {\tilde{\beta }}_{n}, & {\tilde{\beta }}_{n}\in \left[-\frac{r}{2},\frac{r}{2}\right]\\ \frac{r}{2}, & {\tilde{\beta }}_{n}>\frac{r}{2}\\ -\frac{r}{2}, & {\tilde{\beta }}_{n}<-\frac{r}{2} \end{array}$$

In the above Bayesian inference, we maximize the posterior probability to find out the updated formula of the two hyperparameters $\alpha_{0}$ and γ. Then, through these two hyperparameters, we use the expectation maximization criterion to find out the update formula of the angle correction vector hyperparameter β and obtain the final grid error $\beta_{n}$. Finally, we initialize $\alpha_{0}$, γ, and β and keep iterating Formulas (30), (31), and (37) until convergence, so that we can calculate the estimated value of the *K* DOAs as $\theta_{k}=\theta_{n_{k}}+{\tilde{\beta }}_{n_{k}}\;$, where  k=1,2,⋯,K.

The specific flow of the algorithm for iterative EMD interval thresholding and off-grid sparse Bayesian learning is as follows:

(1) Pass the received signal from the array through EMD-IIT to obtain the denoised signal $\hat{X}$.
(2) Construct the off-grid sparse model to obtain the overcomplete sparse dictionary Φ and perform the singular value decomposition of $\hat{X}$ to obtain ${\hat{X}}_{\mathrm{SV}}$.
(3) Initialize the hyperparameters $\alpha_{0}$ and γ for the noise and signal, respectively, and initialize the angle correction vector β, the mean μ, and the variance Σ to zero.
(4) Use Formulas (28) and (29) to solve for the mean and variance, respectively.
(5) Use Formulas (31)–(33) to obtain the updated hyperparameters $\alpha_{0}^{\mathrm{new}}$, $\gamma^{\mathrm{new}}$ and $\beta^{\mathrm{new}}$.
(6) When ${\left\|\gamma^{n}-\gamma^{n-1}\right\|}_{2}/{\left\|\gamma^{n-1}\right\|}_{2}\leq \tau$ or the maximum number of iterations is reached, continue to the next step; if it does not converge, skip to step 4.
(7) Calculate DOA estimates for the target.

## 3. Simulation Analysis

In order to verify the feasibility of the algorithms in this paper, simulation analysis is performed in this section and the estimated performance of the three algorithms is compared. If not specified, the following parameters and initial values were used in the DOA estimation of the objectives: In OGSBI-SVD and EMDIIT-OGSBI, ρ=0.01 and $c=d=1\times 10^{-4}$ are set. $\alpha_{0}=100K/\sum_{t=1}^{K}Var\left\{{\left(Y_{\mathrm{SV}}\right)}_{t}\right\}$, $\gamma =1/MK\sum_{t=1}^{K}\left|A^{H}{\left(Y_{\mathrm{SV}}\right)}_{t}\right|$, and β=0 are initialized. The uniform line array element M=8 is initialized with a grid distance of r=1, $\tau =10^{-3}$ is set, and the maximum number of iterations is 800. The original signal is a frequency band signal with a center frequency of $f_{m}=1000\;\mathrm{Hz}$, $\lambda ={v/f}_{m}$, the sampling frequency is 10 kHz, v=1500 m/s is set as the underwater sound velocity, and the adjacent array element spacing is d=λ/2=0.75 m. The signal-to-noise ratio is calculated as $\mathrm{SNR}=10\mathrm{lg}(P_{s}/P_{n})$, where $P_{s}$ is the signal power, $P_{n}$ is the noise power, and the spatial angle is divided into [−π/2,π/2], and the noise is complex Gaussian white noise. The root mean square error (RMSE) is defined as $\mathrm{RMSE}=\sqrt{\frac{1}{SK}\sum_{s=1}^{S}\sum_{k=1}^{K}{\left({\hat{\theta }}_{k_{s}}-\theta_{k}\right)}^{2}}$, where S denotes the number of Monte Carlo experiments, K is the number of source signals, and ${\hat{\theta }}_{k_{s}}$ denotes the orientation estimate of the kth signal source in the first experiment.

### 3.1. EMD-IIT Denoising Analysis

**Figure 5:**
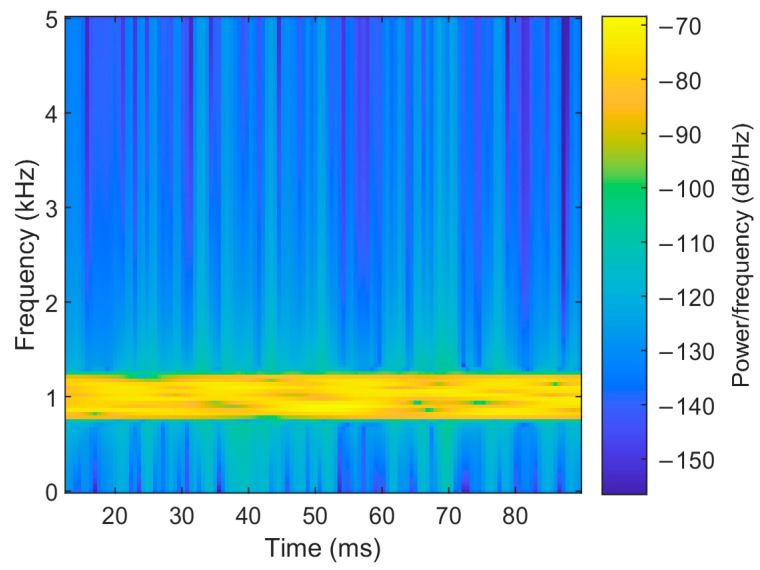
The time–frequency spectrum of the original signal.

**Figure 6:**
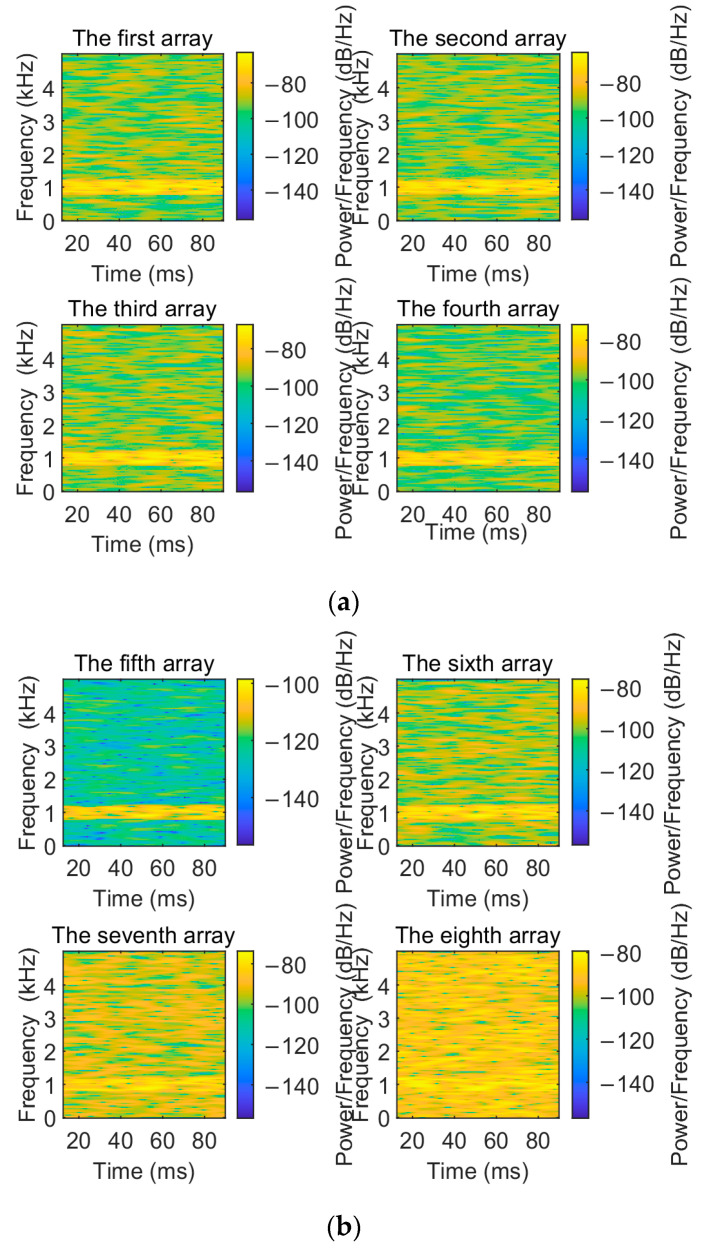
The time–frequency spectrum of each array when the noisy signal is received. (**a**) The time–frequency spectrum of the first to the fourth array when the noisy signal is received. (**b**) The time–frequency spectrum of the fifth to the eighth array when the noisy signal is received.

**Figure 7:**
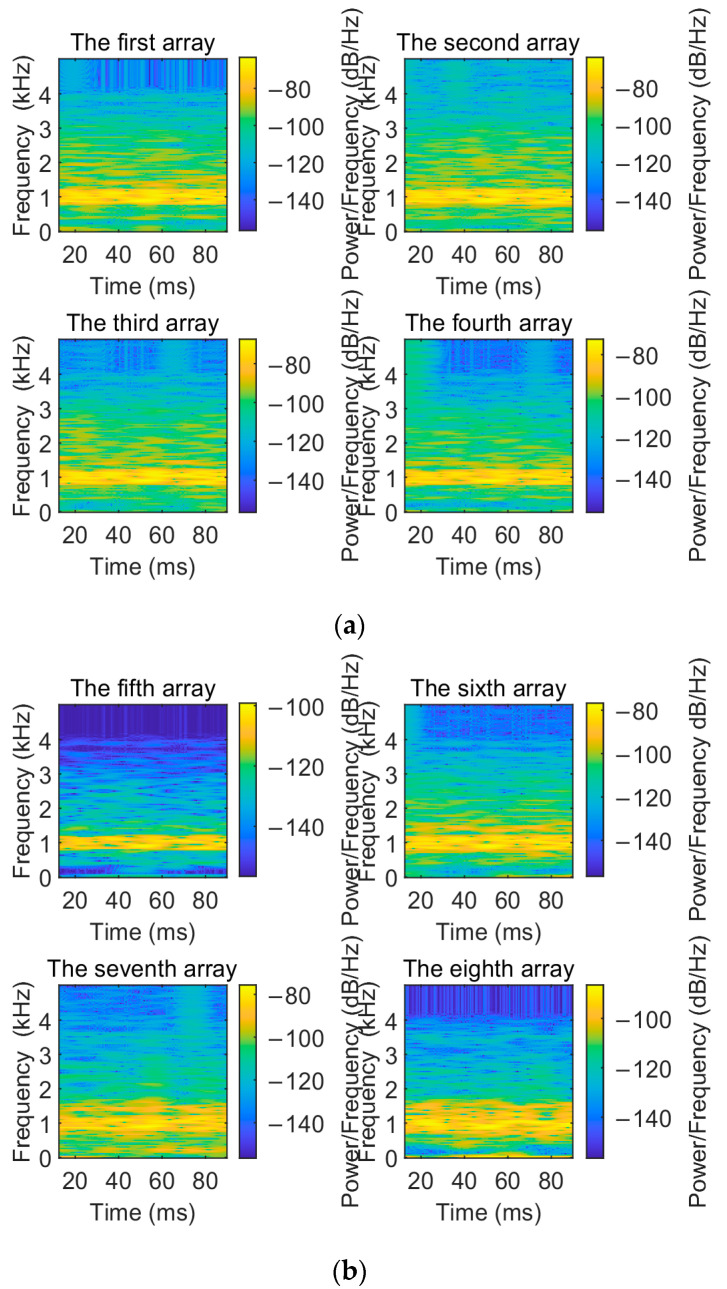
The time–frequency spectrum of each array after EMD-IIT denoising. (**a**) The time–frequency spectrum of the first to the fourth array after EMD-IIT denoising. (**b**) The time–frequency spectrum of the fifth to the eighth array after EMD-IIT denoising.

To visualize the denoising performance of the EMD-IIT algorithm, the noisy signals received by each array element are compared and analyzed with the original signals in this section. The time–frequency spectrum of the original signal is shown in Figure 5. Let the incident directions of the two target signals be [−3.6° 11°]; the number of snapshots is T=1024. The time–frequency spectrum of the received signals of each array element at a signal-to-noise ratio of 5 dB is given in Figure 6. Figure 7 gives the time–frequency spectrum of the received signals of each array element after the EMD-IIT denoising algorithm. We can see that after the EMD-IIT denoising algorithm, a portion of the frequency interference has been effectively removed from the time–frequency spectrum of the array element. This is due to the enhanced noise reduction capability of the EMD-IIT algorithm through multiple noises averaging by the translation-invariant wavelet threshold. The signal-to-noise ratio after denoising and the signal-to-noise ratio before denoising are given in Table 1 for each array element, which further illustrates the good denoising performance of EMD-IIT.

### 3.2. Spatial Power Spectrum Estimation Analysis

**Figure 8:**
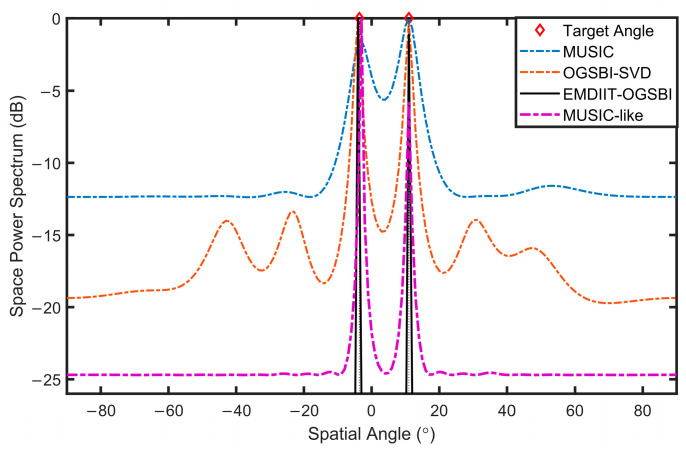
The spatial power spectrum of three algorithms.

To validate the proposed method and highlight its superiority, we compare it under identical conditions with several existing algorithms, including the MUSIC algorithm, the off-grid sparse Bayesian inference algorithm (OGSBI), and the MUSIC-like algorithm. This comparative analysis demonstrates the advantages of our algorithm in terms of performance and accuracy. The orientation of the two target signals is [−3.6° 11°], SNR=0 dB, and the number of snapshots is T=1024. Figure 8 illustrates that the EMDIIT-OGSBI algorithm, as proposed in this paper, is capable of matching the target incident orientation, exhibiting higher spatial–spectral gain and a narrower main flap width. The OGSB-SVD algorithm is able to distinguish the target orientation; however, the main flap width is wider and comprises four pseudo-peaks. The MUSIC algorithm is capable of approximating the target orientation; however, the main flap width is wider, and the peak is not discernible. The MUSIC-like algorithm is able to align with the target orientation; however, its main lobe width is wider, and the spatial gain is inferior in comparison to the algorithm proposed in this paper.

### 3.3. Root Mean Square Error Analysis

#### 3.3.1. RMSE of the Algorithm at Different Numbers of Monte Carlo Trials

**Figure 9:**
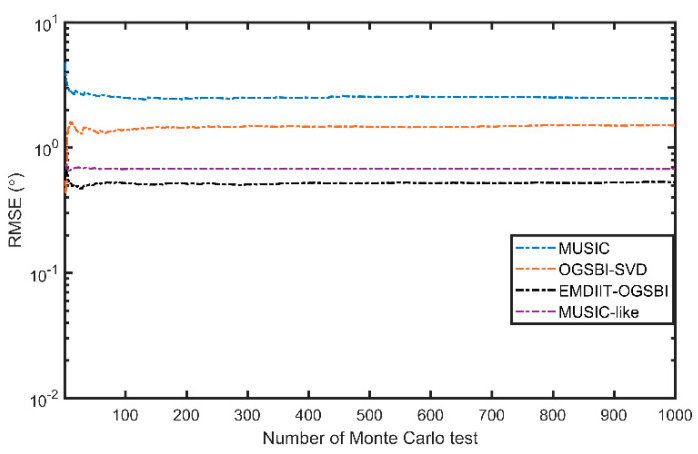
RMSE vs. number of Monte Carlo trials.

Figure 9 shows the variation in RMSE with the number of Monte Carlo trials. The two target orientations in this experiment are [−3.6° 11°], the signal-to-noise ratio is 0 dB, the number of snapshots is 1024, and other parameters are unchanged. It can be seen from the graph that when the number of Monte Carlo trials is less than 100, the RMSE values of the four algorithms converge less, and the DOA estimates of the algorithms have a chance at this time. To avoid this problem, the number of Monte Carlo trials in the following root mean square error analysis is 200.

#### 3.3.2. RMSE of the Algorithm at Different Signal-to-Noise Ratios

**Figure 10:**
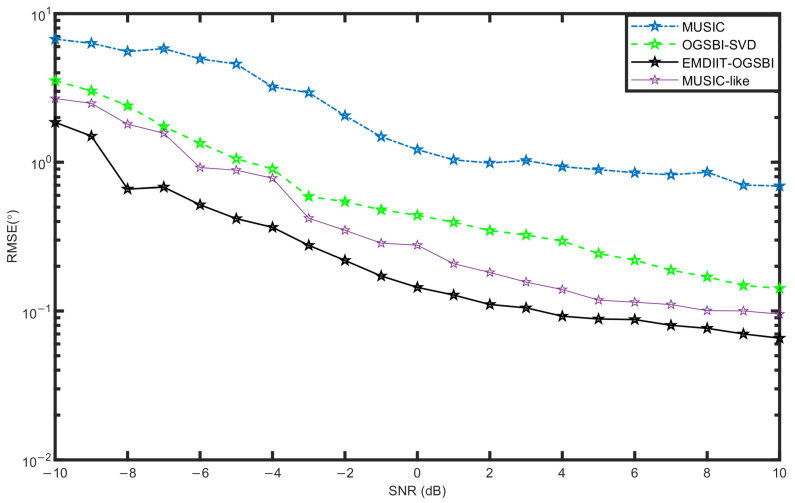
RMSE vs. signal-to-noise ratios.

Figure 10 shows the variation in root mean square error with signal-to-noise ratios for the four algorithms. The number of snapshots is 1024, SNR=−10:1:10 dB, and the two target signal orientations are [−14.3° 6°], respectively. As shown in Figure 9, the MUSIC algorithm has the relatively highest RMSE value at low SNRs, and its orientation estimation performance is limited. The OGSBI-SVD algorithm and the MUSIC-like algorithm have higher RMSE values; however, their performance improves as the signal-to-noise ratio increases, leading to better orientation estimation under the influence of weak noise. In contrast, the EMDIIT-OGSBI algorithm in this paper has a relatively lower RMSE value and a relatively more outstanding DOA estimation accuracy and still has a high DOA estimation accuracy at low signal-to-noise ratios. This is due to the suppression of Gaussian noise by the EMDIIT denoising algorithm, and the singular value decomposition of the off-grid sparse observation matrix also reduces the effect of noise interference.

#### 3.3.3. RMSE of Algorithms at Different Snap Counts

**Figure 11:**
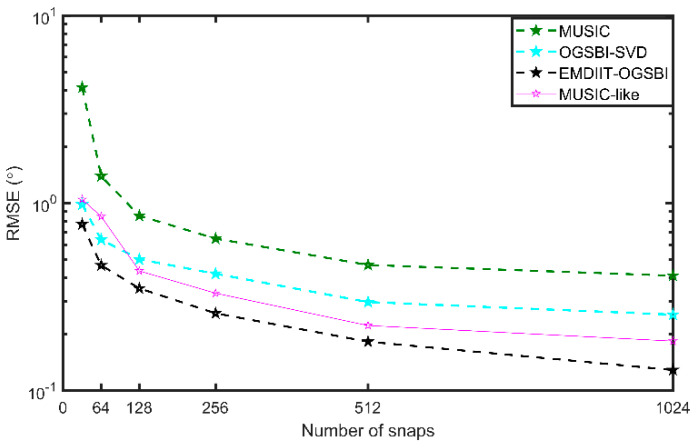
RMSE vs. snaps.

Figure 11 shows a comparison of the RMSEs of the four algorithms for different numbers of snapshots. The range of the number of snapshots is T=[32,64,128,256,512,1024], the signal-to-noise ratio is −3 dB, the two target orientations are [−17.2° 2.4°], and other conditions remain unchanged. As can be seen from Figure 11, the RMSE values of the four algorithms gradually decrease as the number of snapshots increases, while the RMSE values of the proposed algorithm are relatively lower, and the target orientation can be accurately estimated under the low snapshot condition.

#### 3.3.4. RMSE of the Algorithm at Different Grid Distances

**Figure 12:**
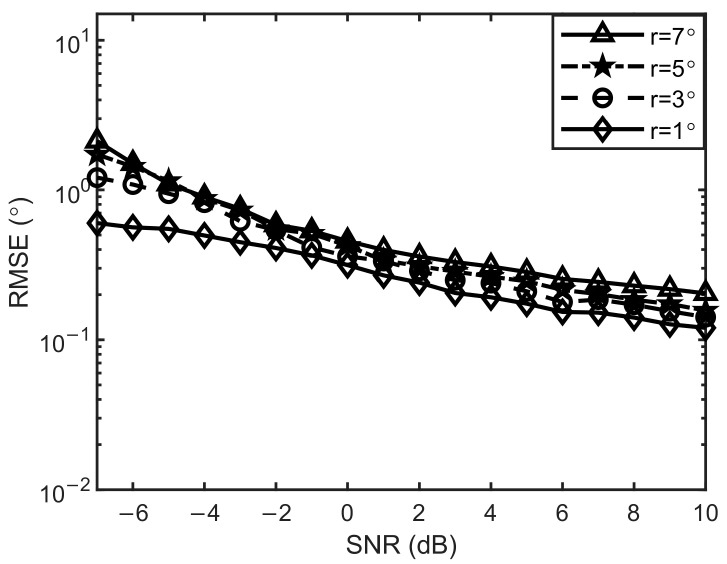
Variation plots of RMSE with signal-to-noise ratios under different grid distances.

Figure 12 shows the RMSE values of the proposed algorithm in this paper as a function of the S/N ratio at grid distances r=[1° 3° 5° 7°]. The two target orientations are [−14.4° 9.5°], the number of snapshots is 1024, the signal-to-noise ratio range is SNR=−7:1:10 dB, and other conditions remain unchanged. As can be seen from Figure 12, the RMSE values of the four different grid distances gradually decrease as the signal-to-noise ratio increases. The finer the grid distance is divided, the smaller the RMSE value is. We can also see that the difference in RMSE between coarse and fine grid distance is insignificant, which shows that the proposed algorithm still has high accuracy in estimating the target orientation under coarse grid distance. It is worth noting that the algorithm in this paper can still maintain a high estimation accuracy even with coarse grid spacing.

### 3.4. Analysis of the Discriminative Probability of Compact Sound Sources

**Figure 13:**
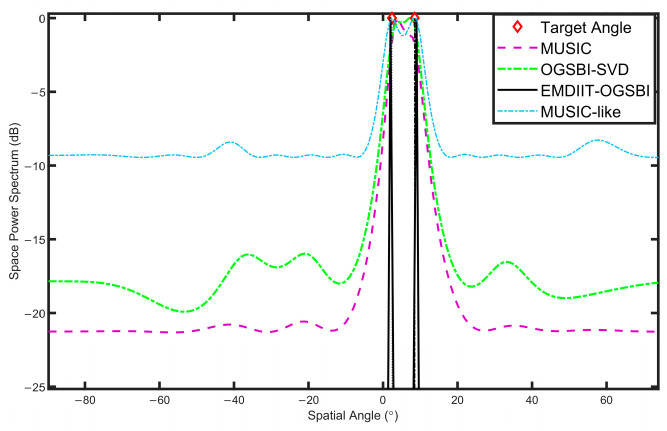
Spatial power spectrum of compact sound source.

**Figure 14:**
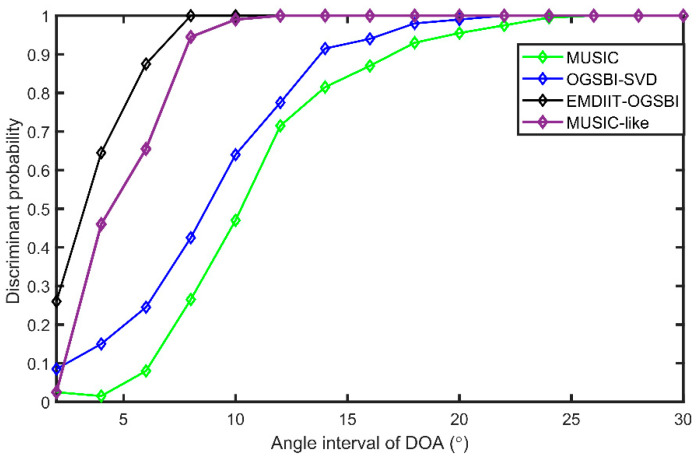
Discriminative probabilities at different DOA intervals.

This section focuses on the analysis of the spatial resolution probability of compact sound sources. Under a uniform line array, the spatial resolution capability of the algorithm in this paper for compact sound sources is analyzed. We set the incident directions of the two target signals as [2.4° 8.5°], the signal-to-noise ratio as 5 dB, the number of snapshots as 1024, and the grid distance as 1°, with other conditions being constant. Figure 13 gives the spatial–spectral estimation plots of the four algorithms for compact sound sources. From Figure 13, it can be seen that compared with the MUSIC, MUSIC-like, and OGSBI-SVD algorithms, the algorithms in this paper have a better spatial resolution for the compact sound sources and have narrower main flap widths and sharper peaks, indicating that the orientation estimation of the compact sound sources also has higher accuracy. To further investigate the discrimination ability of the algorithm proposed in this paper for spatially tight signal DOA estimation, the discrimination probabilities of the three algorithms are analyzed at different DOA intervals with a signal-to-noise ratio of 0 dB with other conditions being constant. The DOAs of the two target signals are defined as $\theta_{1}=u{}^{\circ}$ and $\theta_{2}=(u+\Delta \theta ){}^{\circ}$, and their corresponding spatial power spectrum values are $\zeta_{1}$ and $\zeta_{2}$; the DOA interval Δθ was varied from 2° to 30° in steps of 2°, and 200 Monte Carlo experiments are performed at each DOA interval. The intermediate values of the two target DOAs are set to $\theta_{\mu }=\left(\theta_{1}+\theta_{2}\right)/2$, and $\theta_{\mu }$ corresponds to a spatial power spectrum value of $\zeta_{\mu }$; if $\zeta_{\mu }\leq \left(\zeta_{1}+\zeta_{2}\right)/2$ is satisfied, the discrimination of the compact sound sources is successful. As can be seen from Figure 14, the EMDIIT-OGSBI algorithm in this paper achieves a 100% resolution probability at source intervals Δθ≥8°. The OGSBI-SVD algorithm achieves 100% resolution probability at Δθ=22°; the MUSIC algorithm achieves 100% resolution probability at Δθ=26°; and the MUSIC-like algorithm achieves 100% resolution probability at Δθ=12°. It can be seen that the algorithm has a good spatial resolution of spatially immediate neighboring signals.

### 3.5. Analysis of the Discriminative Probability at Different Signal-to-Noise Ratios

**Figure 15:**
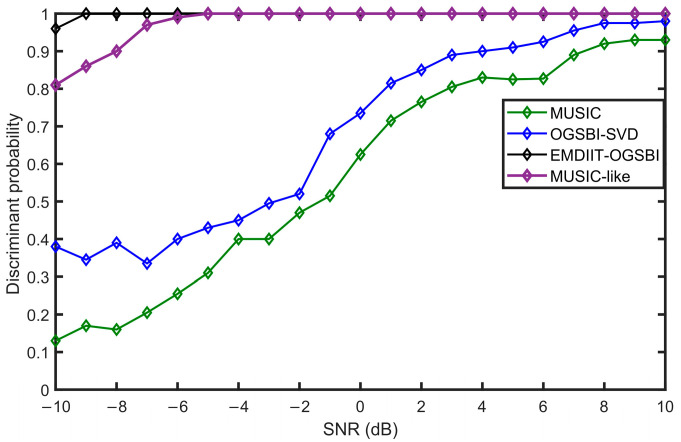
Discriminative probabilities at different signal-to-noise ratios.

To investigate the algorithms’ ability to discriminate between targets at low signal-to-noise ratios, the following simulations are made. The incident directions of the two target signals are randomly generated in the range of [−90° 90°], the separation between them is 14°, and the signal-to-noise ratio range is SNR=−10:1:10 dB, with all other conditions being equal. Figure 15 shows the resolution probabilities of the four algorithms as the signal-to-noise ratio varies. From this figure, it can be seen that the MUSIC and OGSBI-SVD algorithms have a poor resolution of the target at a low S/N ratio, and their resolution ability is improving as the S/N ratio increases. Compared to the MUSIC and OGSBI-SVD algorithms, the MUSIC-like algorithm has better target resolution capability at low signal-to-noise ratios, with its resolution probability reaching 100% at a low SNR of −4 dB. In contrast to the other three algorithms, the proposed EMDIIT-OGSBI algorithm still has a very strong resolution of the target at low SNRs, and the resolution probability reaches 100% only at a low SNR of −9 dB, which effectively shows that the algorithm still has an excellent resolution of the target signal at low SNRs.

### 3.6. Algorithm Runtime Analysis

**Figure 16:**
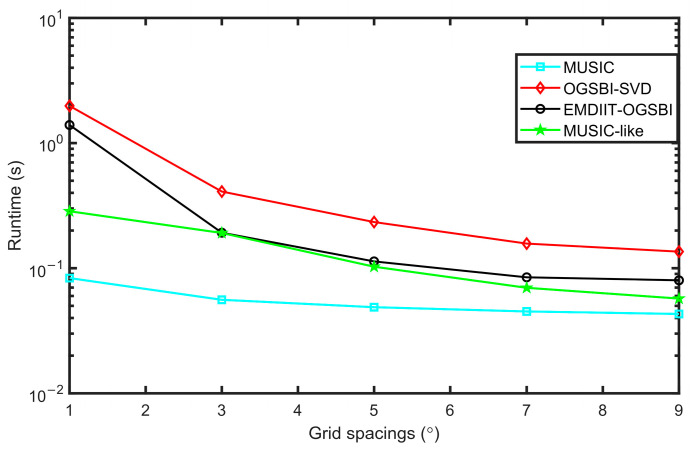
Runtimes at different grid spacings.

Figure 16 shows the comparison of the algorithm running time of the four algorithms with different grid spacing. SNR = 5 dB was set, the number of snaps is 1024, and the two target incidence directions are [−13.4° 5.6°], respectively. We can see that the running time of the four algorithms decreases as the grid spacing increases. The MUSIC algorithm has the shortest runtime because it does not involve iterative operations; however, this also results in its poor orientation estimation performance, as discussed earlier. The MUSIC-like algorithm requires the calculation of fourth-order cumulants, which increases its runtime. The EMDIIT-OGSBI algorithm includes iterative operations not only in the noise reduction process but also in the azimuth angle estimation, leading to a longer runtime than the MUSIC algorithm. Nevertheless, its runtime is still shorter than that of the OGSBI-SVD algorithm. This is because, in the EMDIIT-OGSBI algorithm, the EMDIIT method effectively reduces the impact of background noise, significantly decreasing the number of iterations required by OGSBI. Additionally, singular value decomposition is employed to reduce the signal’s dimensionality and discard unnecessary signal matrices, further reducing the runtime.

## 4. Validating Algorithms with Sea Trial Data

**Figure 17:**
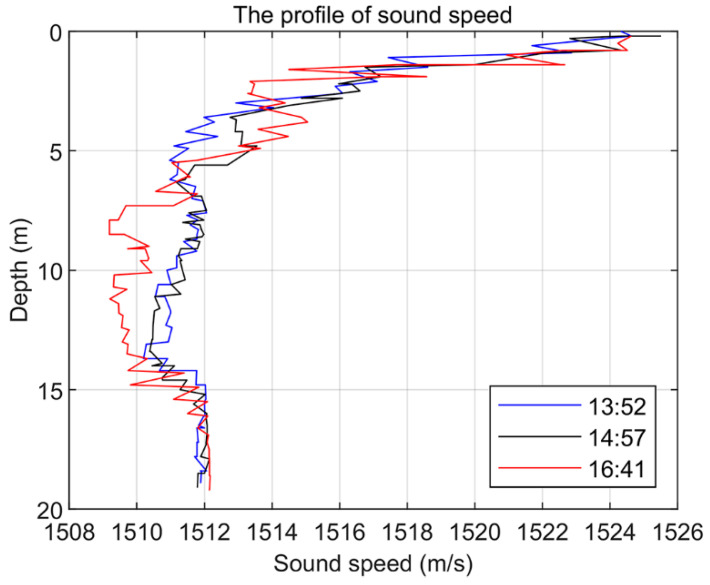
The profile of sound speed.

**Figure 18:**
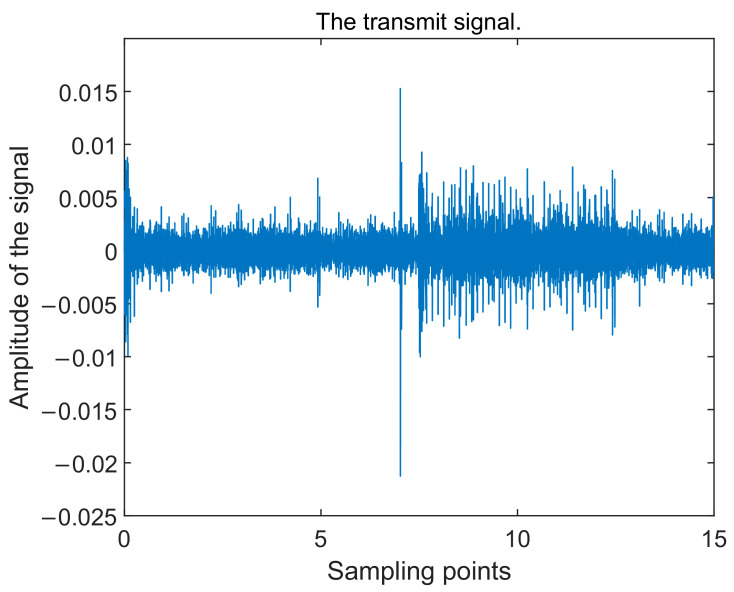
The transmitted signal.

**Figure 19:**
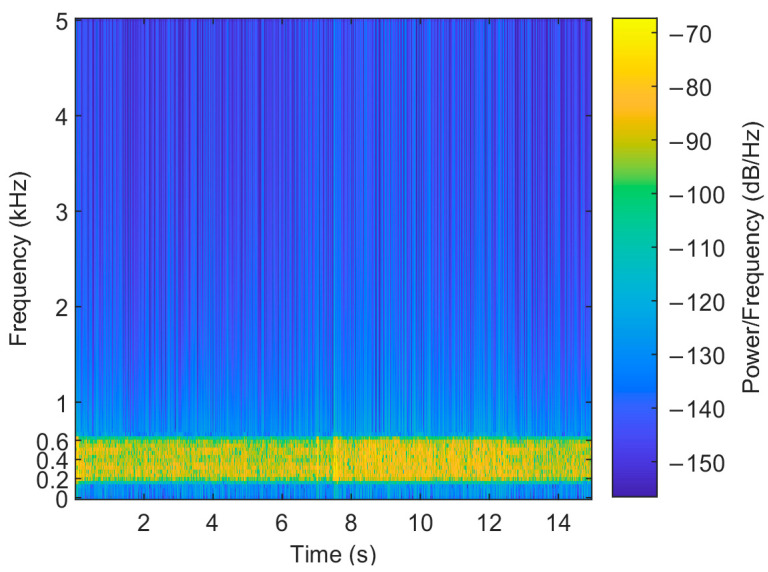
The time–frequency spectrum of the sound source.

**Figure 20:**
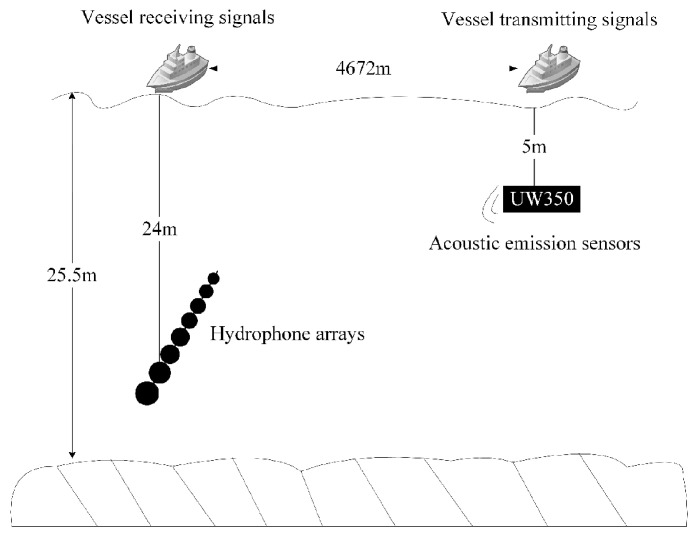
Sea trial deployment diagram.

The data obtained from a sea trial experiment conducted in a sea area are used to verify the performance of the algorithm. The experimental sea area on that day was calm in terms of ocean noise, with no other passing vessels on the surface and low wind speeds. The sound velocity profiles of the sea surface on that day are shown in Figure 17, which were measured at 13:52 p.m., 14:57 p.m., and 16:41 p.m. In the sea trial, the sound source transmitting equipment UW350 was lifted at a depth of 5 m and the transmitting signal was a broadband long pulse signal of 200–600 Hz, with a signal length of 15 s and a sampling frequency of 10 kHz. The transmitted signal is shown in Figure 18. The time–frequency spectrum of the sound source is shown in Figure 19. The number of hydrophone array elements is eight, the array element spacing is half a wavelength, and the experimental sea depth is 25.5 m. The above equipment deployment depth and seawater depth are measured by the depth sensor. The schematic layout of the sea trial is shown in Figure 20.

**Figure 21:**
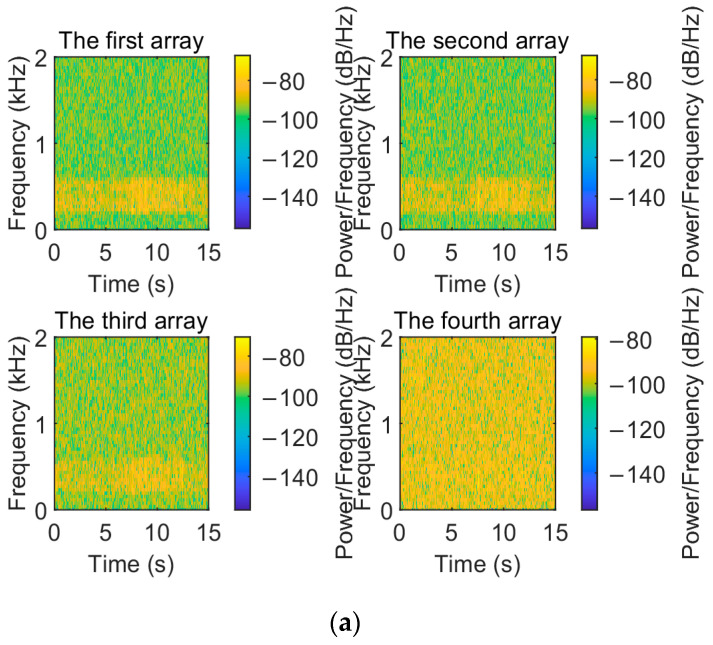

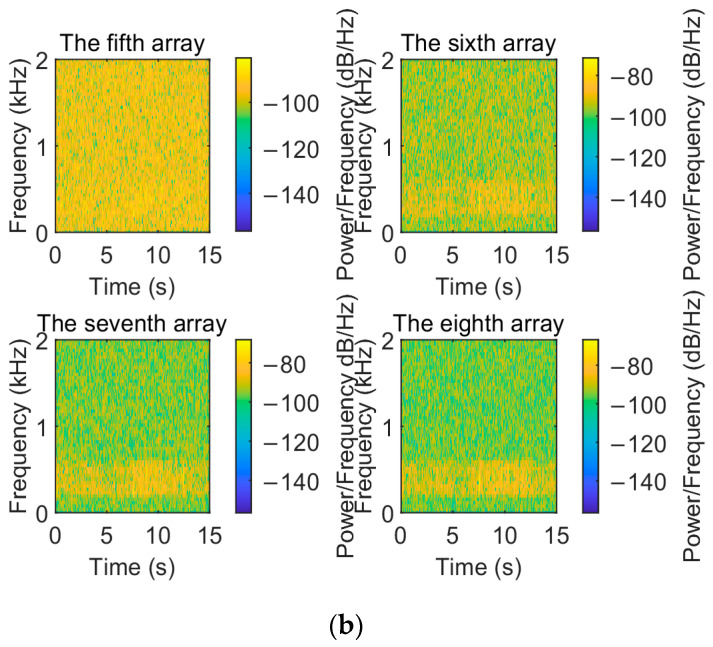
The time–frequency spectrum of each array at 14:57 p.m. (**a**) The time–frequency spectrum of the first to the fourth array. (**b**) The time–frequency spectrum of the fifth to the eighth array.

**Figure 22:**
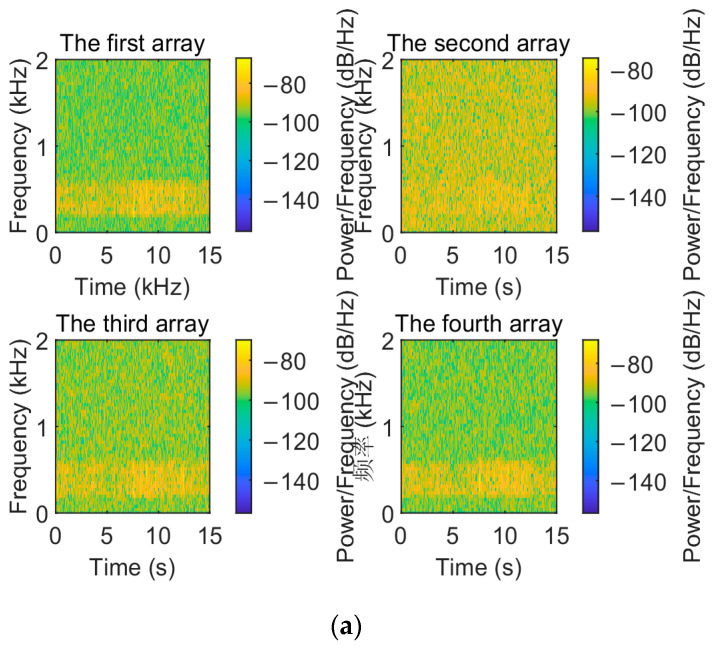

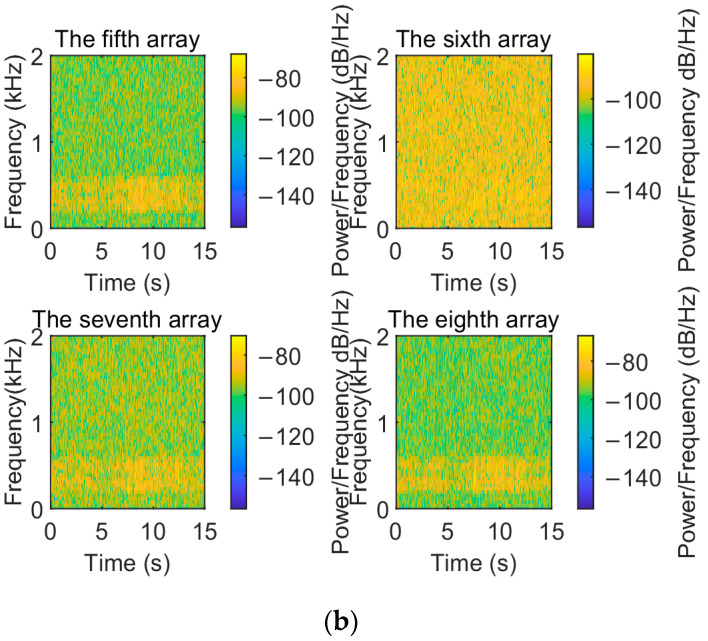
The time–frequency spectrum of each array at 16:41 p.m. (**a**) The time–frequency spectrum of the first to the fourth array. (**b**) The time–frequency spectrum of the fifth to the eighth array.

**Figure 23:**
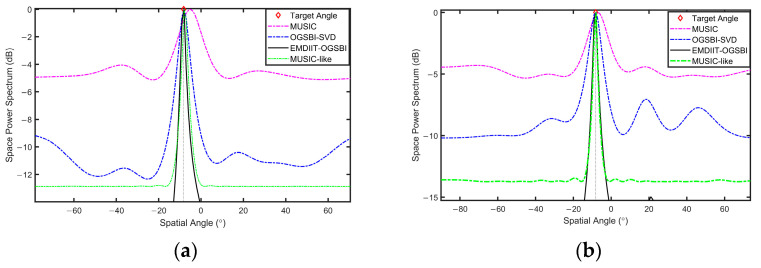
Estimation of the spatial power spectrum at the second position. (**a**) The snapshot count is 512. (**b**) The snapshot count is 1024.

**Figure 24:**
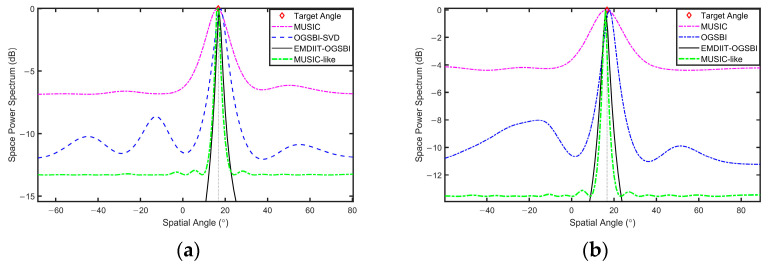
Estimation of the spatial power spectrum at the third position. (**a**) The snapshot count is 512. (**b**) The snapshot count is 1024.

DOA verification of the target was carried out by selecting data from two different locations from the sea trials. The target orientations were [−8.2° 16.7°], which were placed at 14:57 p.m. and 16:41 p.m., respectively. Figure 21 and Figure 22 show the signal’s time–frequency spectrum of the eight array elements collected at two time periods, respectively. It can be seen that the noise of the day interferes with the original signal. The results of DOA spatial spectrum estimation at different target orientations are given in Figure 23 and Figure 24, respectively. In the orientation estimation of the sea trial data, the number of snapshots chosen is 512 and 1024. From these figures, it can be seen that the MUSIC algorithm can roughly distinguish the target orientation, has some deviation in the target orientation estimation, has a wide main flap width and poor spatial power spectrum gain, and the OGSBI-SVD algorithm can approximate the target orientation, with a relatively narrow width of the main flap and a more pronounced peak. However, there are pseudo-peaks in the spatial power spectrum, which will seriously affect the discrimination of the true orientation; the MUSIC-like algorithm is relatively stable and is capable of distinguishing target direction angles with a narrower main lobe width. However, it exhibits poor spatial gain, whereas the EMDIIT-OGSBI algorithm in this paper has a narrower main flap width and higher spatial–spectral gain, which can estimate the target orientation more accurately. In summary, the DOA estimates of the four algorithms are approximately equal to the actual deployed orientation. In comparison, both the EMDIIT-OGSBI algorithm and the MUSIC-like algorithm demonstrate high accuracy at low snapshot counts, are less affected by the number of snapshots, and exhibit better robustness.

To better compare the performance of the four algorithms for the orientation estimation of the sea trial data, 200 Monte Carlo experiments are conducted on the sea trial experimental data of the three positions, and the number of snapshots is chosen as 1024, and the mean value and root mean square error of the DOA estimation results are recorded in Table 2. The estimation results of the algorithm in this paper are closer to the target bearing and have a stronger suppression ability to the background noise interference of the ocean, which is generally consistent with the numerical simulation analysis.

## 5. Conclusions

This paper investigates the application of EMD-IIT-based denoising with off-grid sparse Bayesian learning algorithms for hydroacoustic direction-of-arrival (DOA) estimation. The proposed algorithm effectively reduces noise, improves target resolution, and enhances estimation accuracy.

The EMDIIT-OGSBI algorithm demonstrates strong robustness, shorter runtime, and reduced sensitivity to grid spacing and the number of snapshots compared to conventional algorithms. It maintains high resolution for target signals and neighboring signals in the presence of strong noise interference. Overall, the algorithm addresses the challenges of low precision and poor resolution in target DOA estimation under low signal-to-noise ratios, making it a relevant and beneficial approach in hydroacoustic signal processing.

## Figures and Tables

**Table 1:** SNR of each array before and after denoising.

Array	Before Denoising/(dB)	After Denoising/(dB)
The 1st	5	14.067
The 2nd	5	14.414
The 3rd	5	13.863
The 4th	5	14.242
The 5th	5	13.844
The 6th	5	14.397
The 7th	5	13.840
The 8th	5	14.071

**Table 2:** Mean and root mean square errors of DOA estimate.

Position	Performance	EMDIIT-OGSBI	OGSBI-SVD	MUSIC	MUSIC-Like
The first position	Mean/(°)	−7.925	−8.986	−5.930	−8.726
	RMSE/(°)	0.402	1.499	3.620	0.564
The second position	Mean/(°)	16.687	15.924	14.650	16.545
	RMSE/(°)	0.154	0.979	4.680	0.251

## Data Availability

Data are not available due to privacy or ethical restrictions.
